# Prevalence of Chronic Hepatitis B and Hepatitis C among First Time Blood Donors in Northeast Bosnia and Herzegovina: An Estimate of Prevalence in General Population

**DOI:** 10.5812/kowsar.1735143X.716

**Published:** 2011-08-01

**Authors:** Jasminka Petrovic, Nermin N. Salkic, Sead Ahmetagic, Vildana Stojic, Slavica Mott-Divkovic

**Affiliations:** 1 Department of Infectious Diseases, University Clinical Center Tuzla, Tuzla, Bosnia and Herzegovina; 2 Department of Gastroenterology and Hepatology, University Clinical Center Tuzla, Tuzla, Bosnia and Herzegovina; 3 Department of Transfusion Medicine, University Clinical Center Tuzla, Tuzla, Bosnia and Herzegovina

**Keywords:** Hepatitis B, Hepatitis C virus, Epidemiology, Blood donors, Population, Bosnia and Herzegovina

## Abstract

**Background:**

Data on the epidemiology of hepatitis B and C in Bosnia and Herzegovina (B&H) are lacking.

**Objectives:**

To assess the prevalence of hepatitis B surface antigen (HBsAg) and anti-hepatitis C virus (anti-HCV) in blood samples of first time blood donors in a well-defined region of B&H. Our secondary goal was to estimate the prevalence of HBsAg and anti-HCV in the general population of the same region.

**Patients and Methods:**

We evaluated 8196 blood samples for the presence of HBsAg and/or anti-HCV, adjusted for differences in gender, and used the ratio estimation method to determine the prevalence in the general population.

**Results:**

We analyzed 1263 (15.4%) female and 6933 (84.6%) male blood donors (male-to-female ratio: 5.49 to 1). The adjusted prevalence of HBsAg among blood donors was 0.787% (95% CI = 0.535-1.038), while the prevalence of anti-HCV was 0.267% (95% CI = 0.016-0.519). There was no difference in the prevalence of HBsAg or anti-HCV between men and women. We estimate that the prevalence of HBsAg and anti-HCV in the general population is 1.057% to 1.535% and 0.29% to 0.89%, respectively.

**Conclusions:**

The prevalence of HBsAg and anti-HCV among blood donors suggests that our region has low endemicity for both hepatitis B and hepatitis C.

## 1. Background

Viral hepatitis B and hepatitis C are significant global health issues for industrialized and developing countries. It is estimated that there are 2 billion people who have been exposed to hepatitis B worldwide, with 350 million suffering from chronic infection [1]. The statistics for hepatitis C are hardly better - it is estimated that there are more than 200 million people who are chronically infected throughout the world [2]. Hepatitis B is estimated to result in 563,000 deaths annually versus 366 000 deaths for hepatitis C [3]. Data on the epidemiology of hepatitis B and C in Bosnia and Herzegovina (B&H) are sparse. There are some reports of the prevalence of hepatitis B among smaller groups of first time blood donors and the prevalence of hepatitis C in special groups of patients [4][5][6][7]. However, there are no accurate data regarding the numbers of those who have been exposed and chronically infected in the general population in B&H.

## 2. Objectives

We aimed to assess the prevalence of hepatitis B surface antigen (HBsAg) and anti-hepatitis C virus (anti-HCV) in blood samples of all first time blood donors who resided in a very well-defined region of northeast B&H in 2009. Our secondary goal was to estimate the prevalence of HBsAg and anti-HCV in the general population of this region.

## 3. Patients and Methods

### 3.1. Settings

Since the end of the Bosnian War (1992-1995), Bosnia and Herzegovina has comprised 2 entities - the Federation of Bosnia and Herzegovina and the Republic of Srpska. The Federation of Bosnia and Herzegovina consists of 10 cantons, one of which is the Tuzla Canton, a well-defined region in the northeast of Bosnia and Herzegovina. The estimated population in the region, as of December 31, 2010, is 496,280 (Federal Office of Statistics, Federation of Bosnia and Herzegovina, Sarajevo). University Clinical Center Tuzla is a regional hospital center that serves as a secondary and tertiary referral center for the region. Blood donation in B&H is exclusively voluntary, without the possibility of financial compensation for donations.

### 3.2. Patients and Methods

In 2009 (January 1st to December 31st, 2009), we evaluated approximately 10,000 voluntary blood donors at the Department of Transfusion Medicine in University Clinical Center Tuzla (Tuzla, B&H). All volunteers were obliged to satisfy the hospital's criteria, which included: age over 18 and less than 65, body weight over 50 kg, normal body temperature, absence of clinical signs of an acute infective response, normal systolic and diastolic blood pressure, normal pulse and respiration rate, and absence of serious chronic condition (eg, chronic heart failure, diabetes mellitus with complications). Inclusion criteria were first time blood donations and place of residence in the Tuzla Canton.

Ultimately, we analyzed 8196 blood samples from the same number of blood donors, which represents 1.651% of the population of Tuzla Canton. We also recorded the age and gender of each blood donor. All blood samples were routinely screened for transfusion-transmitted diseases per established screening procedures and tests. Therefore, all blood samples were tested for HBsAg and anti-HCV by chemiluminescent microparticle immunoassay (CMIA) on the Architect i2000 system (Abbott Laboratories, USA). Every sample that was positive in the initial screen was retested to confirm seropositivity. Markers of exposure to hepatitis B (anti-HBsAg and anti-HBcAg) are not routinely measured in our blood bank. The study protocol was approved by the University Clinical Center Tuzla Ethical Committee.

### 3.3. Statistical Analysis

All tests were performed using SPSS 15.0 (SPSS Inc, USA) and Excel 2007 (Microsoft, USA). Descriptive statistics were used to determine the baseline characteristics. We used student's t-test to compare quantitative variables and chi-square test to compare categorical variables. The prevalence of seropositivity for HBsAg and anti-HCV in blood donors was expressed as the percentage of seropositive samples. We corrected for differences in gender (predominantly males in the sample) per a standard method [8][9]. The prevalence of seropositivity for HBsAg and anti-HCV was standardized based on the yearly (December 31, 2009) population estimates in our region from the Federal Office of Statistics (Federation of Bosnia and Herzegovina, Sarajevo). Gender-standardized incidence rates were calculated using population weights for Tuzla Canton in the age group 18-65 years (Males: 48.9325; Females: 51.0674). 95% confidence intervals (95% CI) of prevalence rates were estimated assuming a Poisson distribution of the cases. We used the ratio estimation method to estimate the prevalence of HBsAg and anti-HCV in the general population, as used for the prevalence of cocaine use [10]. The method entails a calculation of the ratio between known prevalences in 2 populations (i.e. blood donors and general population). This ratio is then used to estimate the prevalence in a population, based on the knowledge of prevalence in another population (i.e. calculating the prevalence in a general population based on the prevalence in blood donors). We reviewed several studies from countries with data on the prevalence of hepatitis B and hepatitis C in general population and blood donors, and using the ratio estimation method, we calculated estimated the prevalence of HBsAg and anti-HCV in general population in Tuzla Canton. A statistical level of 95% (P < 0.05) was considered significant for all tests.

## 4. Results

**Figure 1:**
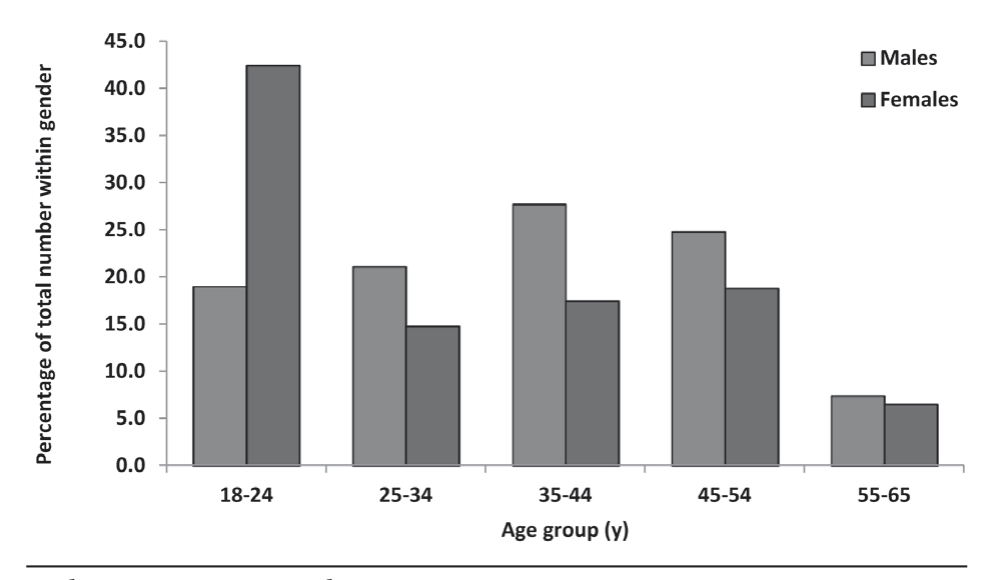
Distribution of the First Time Blood Donors According to Age And Gender. Tuzla Canton. Bosnia and Herzegovina. year 2009

We analyzed 8196 blood samples from 6933 (84.6%) male blood donors. The male-to-female ratio was 5.49 to 1. The mean age ± SD in the entire sample was 36.69 ± 12.21 years, ranging from 18 to 69 years. Male blood donors (37.43 ± 11.81 years) were aged an average of 4.81 older (95% CI = 4.09-5.53) than female donors (32.63 ± 13.49) (t = 13.009; df = 8194; P < 0.001). The distribution of ages and genders is shown in Figure 1.

### 4.1. Prevalence of HBsAg Among Blood Donors

**Figure 2:**
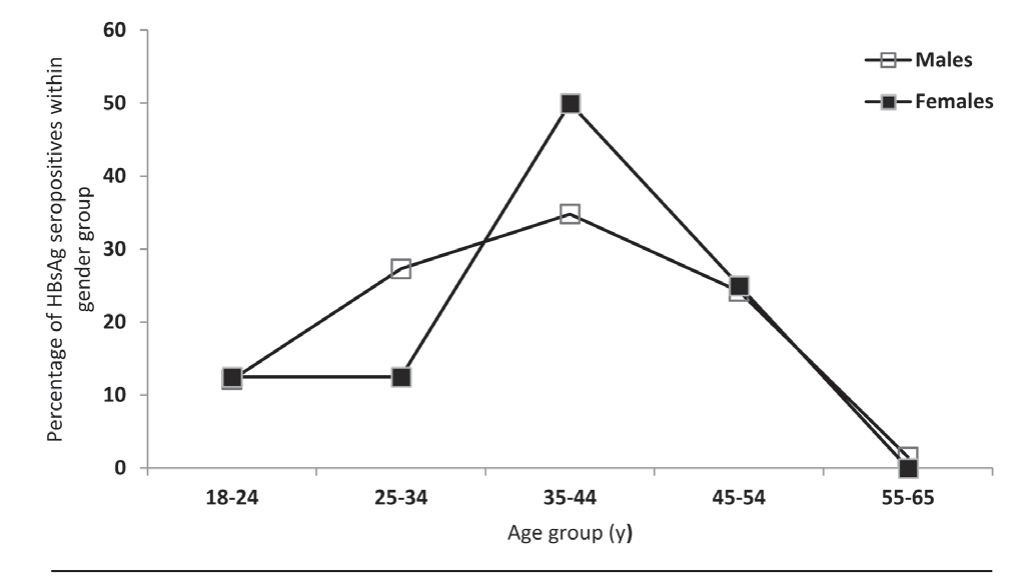
Distribution of HBsAg Seropositivity Among First Time Blood Donors According to Age and Gender. Tuzla Canton. Bosnia and Herzegovina. year 2009

We identified 74/8196 HBsAg-seropositive samples, which corresponded to a crude prevalence of 0.903% (95% CI = 0.7-1.1). There were 66/6963 HBsAg-positive samples among men, consistent with a crude prevalence of 0.947% (95% CI = 0.72-1.18). We detected 8/1263 HBsAg-seropositive samples among female blood donors, corresponding to a crude prevalence of 0.633 (95% CI = 0.19-1.07). There was no significant difference in the frequency of HBsAg seropositivity between males and females (X2 = 1.186; df = 1; P = 0.35). After standardizing by population weights of Tuzla Canton, the corrected and standardized prevalence of the entire sample was 0.787% (95% CI = 0.535-1.038). The percentages of HBsAg-seropositive samples by age and gender are presented in Figure 2.

### 4.2. Prevalence of Anti-HCV Among Blood Donors

**Figure 3:**
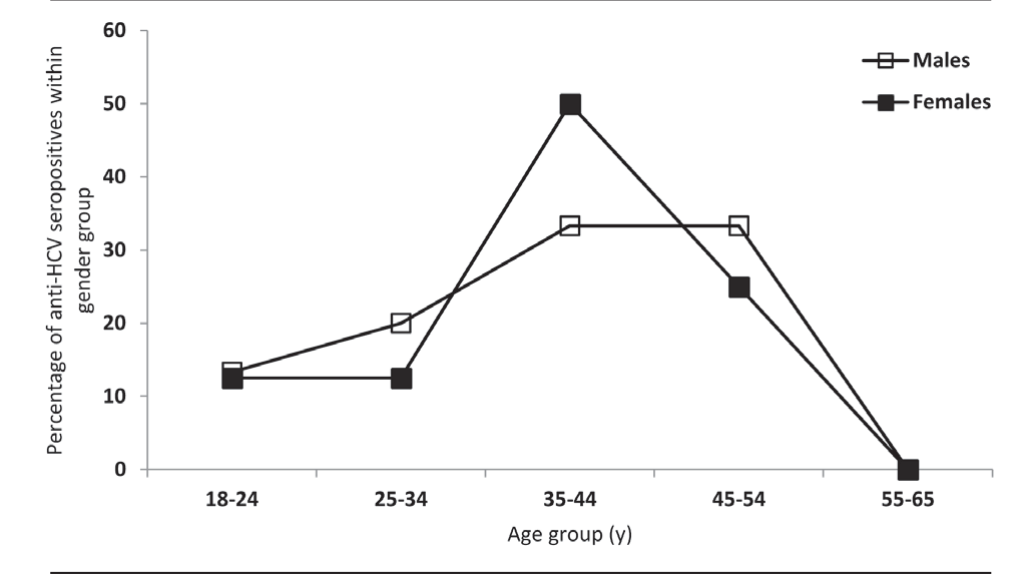
Distribution of Anti-HCV Seropositivity Among First Time Blood Donors According to Age and Gender. Tuzla Canton. Bosnia and Herzegovina. year 2009

We detected 19/8196 anti-HCV-seropositive blood samples, equivalent to a crude prevalence of 0.232% (95% CI = 0.13-0.33). Among men, we detected 15/6963 anti-HCV seropositive cases and 4/1263 such cases among women, which corresponded to a crude prevalence of 0.215% (95% CI = 0.11-0.33) and 0.317% (95% CI = 0.01-0.63), respectively. This difference in ratio of anti-HCV seropositive blood samples between male and female blood donors was not significant (X2 = 0.476; df = 1; P = 0.71). After standardization and correction, we calculated a corrected prevalence of 0.267% (95% CI = 0.016-0.519). The percentage of anti-HCV seropositive samples by age and gender is presented in Figure 3.

### 4.3. Estimated Prevalence of HBsAg and Anti-HCV Seropositivity within the general population of Tuzla Canton

Using the ratio estimation method described above, we assessed the prevalence of HBsAg and anti-HCV seropositivity within the general population of Tuzla Canton. The reported prevalence of HBsAg among blood donors and the general population in various countries is presented in Table 1. We calculated the ratio between the prevalence of HBsAg in the general population and that in blood donors for each country. As seen in Table 1, the ratios ranged from 1.17 to 1.70. Based on these ratios and according to our calculated prevalence among blood donors, we estimated a prevalence of HBsAg in the general population of Tuzla Canton of 1.057% to 1.535%. Similarly, we calculated the ratios of the prevalence of anti-HCV seropositivity between the general population and blood donors by country. As seen in Table 1, the ratios ranged from 1.25 to 3.85. Based on these ratios and according to our calculated prevalence among blood donors, we estimated a prevalence of anti-HCV in the general population of Tuzla Canton of 0.29% to 0.89%.

**Table 1:** Ratios of Country Prevalence of HBsAg and Anti-HCV Seropositivity in General Population and Blood Donors

Country	**Study Report**	**Prevalence Among Blood Donors, %**	**Prevalence Among General Population, %**	**Ratio**
**HBsAg seropositivity**				
England	Soldan K et al., 2003 [17]	0.316	-	1.17
	Gay NJ et al., 1999 [18]	-	0.37	
India	Bhattacharya P et al., 2007 [19]	1.66	-	1.45
	Batham A et al., 2007 [20]	-	2.4	
Greece (Crete)	Koulentaki M et al., 1999 [21]	0.401	0.6	1.5
	Durro V et al., 2010 [22]	6.7	-	1.21
Albania	Kondili LA et al., 2007 [23]	-	8.1	
Croatia	Grgicevic D et al., 2000 [11]	0.341	0.4	1.17
Turkey	Acar A et al., 2010 [24]	1.76	-	1.70
	Pirnar A et al., 1976 [25]	-	3	
**Anti-HCV seropositivity**				
England	Soldan K et al., 2003 [17]	0.16	0.2	1.25
India	Bhattacharya P et al., 2007 [19]	0.35	0.87	2.49
Greece	Koulentaki M et al., 1999 [21]	0.382	-	3.27
	Goritsas C et al., 2000 [10]	-	1.25	
Croatia	Grgicevic D et al., 2000 [11]	0.26	1	3.85

## 5. Discussion

Data on the epidemiology of hepatitis B and C in B&H are lacking. Prior to 2007, the prevalence of hepatitis B in the general population in B&H was estimated by extrapolating the prevalence in neighboring countries. Hence, it was proposed that B&H belonged to a region of intermediate endemicity. In 2007, our group investigated 716 first time blood donors in a separate study, calculating a prevalence of HBsAg positivity of 3.6% [7]. However, this figure must be considered cautiously, due to the small sample. Additionally, we recruited all persons who donated blood during a single month, regardless of their place of residence. The prevalence of HBsAg among blood donors of 0.787% presenting the current study is more reliable and, as seen in Table 1, is much closer to the figures in Croatia, a similar, neighboring country [11]. We were not able to determine the prevalence of HBsAg among blood donors in Serbia (another neighboring country), although the recently separated, underdeveloped region of Kosovo has a significantly higher prevalence of 4.2% [12]. The number of hepatitis B-exposed blood donors should be evaluated, but our Department of Transfusiology does not routinely screen blood for anti-HBsAg or anti-HBcAg. This practice must be changed due to the small but realistic danger of occult hepatitis B infection in HBsAg-negative blood donors [13][14].

Epidemiological reports on hepatitis C prevalence in B&H have dealt primarily with either special or high-risk groups [4][5]. A report from 2006 used a smaller sample of 2000 blood donors as a control group and reported an anti-HCV prevalence of 0.2% [5]. We calculated a prevalence of 0.267%, similar the 0.26% rate reported in Croatia [11]. Additionally, a study from Kosovo reported a similar prevalence of anti-HCV of 0.3% in blood donors [12]. Epidemiological studies of blood donors are frequently used as a surrogate for general population studies on the epidemiology of hepatitis B and C, because an epidemiological assessment of general population is difficult to perform due to the required sample size and recruitment problems. Financial requirements for a survey of such magnitude are another obstacle. On the other hand, donors willingly donate their blood, which is routinely and conveniently screened for hepatitis B and C, allowing for quick sample collection and analysis. Yet, the estimated prevalence of hepatitis B and C in blood donors is nearly always below the actual prevalence in the general population. Blood donors, especially volunteer blood donors, are usually a healthier segment of population and are actively screened prior to donation, which may be reasons for the lower rates. Many countries either conduct nationwide epidemiological surveys (fortunate countries) or combine several regional surveys (less fortunate countries) and extrapolate the overall prevalence, or they do not conduct any surveys on the general population at all; some nations investigate populations that are regularly screened and are close to the general population, such as blood donors (the least fortunate countries). However, the estimated prevalence of a general population is rarely (if ever) based on a particular method of calculation-it is based primarily on speculation. Our study is an attempt to use an established method in a new field and avoid guesswork.

In situations in which it is not easy to perform a population-based study, the ratio estimation method can be used cautiously to estimate prevalence; this method has been successfully used to determine the prevalence of cocaine use in a situation in which it was not possible to conduct a field study [15]. We used the same method to calculate the ratios of prevalence of HBsAg and anti-HCV in the general population and blood donors in several countries. The estimated prevalence of HBsAg seropositivity in the general population in our area of B&H was 1% to 1.5%, suggesting that our country belongs to a group of nations with low endemicity for hepatitis B. With regard to hepatitis C, the estimated prevalence of anti-HCV in the general population of our region ranges from 0.3% to 0.9%, possibly toward the lower end. These values reflect a region with a lower prevalence of hepatitis C and are similar to those in reports on surrounding countries. In support of our estimate, a study calculated a 0.4% prevalence of anti-HCV among 1699 health care workers [5], a well-established risk group with a minor to modest risk for hepatitis C [16].

These numbers must be cautiously interpreted, because they are merely an estimate and do not substitute for a real assessment. Vaccination programs against HBV have existed in our country since 2001, but those born before 2001 are infrequently vaccinated. As noted, blood donors are usually healthier segments of a population and probably less prone to risky behaviors and lifestyles that lead to hepatitis infection. Moreover, blood donors are aged 18 to 65, rendering a substantial proportion of the population uninvestigated. These are merely some of the potential confounders that need to be taken into account. Nevertheless, until there is a population-based survey, this is currently the best estimate. We are, however, convinced that true prevalence lies within the intervals above. The study would have been more reliable if we had included more blood donors - possibly over 2 or 3 years. Yet, such a change would not have affected the prevalence, because we evaluated 1.65% of the general population in our region.

In conclusion, the prevalence of HBsAg and anti-HCV among blood donors suggests that our region has low endemicity for hepatitis B and hepatitis C. Accurate epidemiological surveys must be performed to determine the true prevalence of hepatitidem in the general population.
